# Efficient detection of Zika virus RNA in patients’ blood from the 2016 outbreak in Campinas, Brazil

**DOI:** 10.1038/s41598-018-22159-2

**Published:** 2018-03-05

**Authors:** Carla Cristina Judice, Jeslin J. L. Tan, Pierina Lorencini Parise, Yiu-Wing Kam, Guilherme Paier Milanez, Juliana Almeida Leite, Leonardo Cardia Caserta, Clarice Weis Arns, Mariangela Ribeiro Resende, Rodrigo Angerami, Eliana Amaral, Renato Passini Junior, André Ricardo Ribas Freitas, Fabio Trindade Maranhão Costa, Jose Luiz Proenca-Modena, Lisa F. P. Ng, The Zika-Unicamp Network, Glaucia Maria Pastore, Helaine Maria Besteti Pires Mayer-Milanez, Carolina C. Ribeiro-do-Valle, Roseli Calil, Maria Laura Costa, João Renato Bennini Junior, Giuliane Jesus Lajos, Márcia Teixeira Garcia, Kleber Yotsumoto Fertrin, Maria Luiza Moretti, Marcos Tadeu Nolasco da Silva, Ana Carolina Coan, Maria Francisca Colella-Santos, Andrea Paula Bruno von Zuben, Marco Aurélio Ramirez Vinolo, Rodrigo Ramos Catharino, Gabriela Mansano do Nascimento, Matheus Martini, Ana Paula de Moraes, Ana Lucia Rodrigues Soledade, Daniel Augusto de Toledo Teixeira, Évellyn Ribeiro de Morais, Felipe Rebelo Santos, Monique Fontana

**Affiliations:** 1 Laboratory of Tropical Diseases–Department of Genetics, Evolution and Bioagents, Institute of Biology, University of Campinas (Unicamp), Campinas, São Paulo, Brazil; 2 Singapore Immunology Network, Agency for Science, Technology and Research (A*STAR), Singapore, Singapore; 3 Laboratory of Emerging Viruses–Department of Genetics, Evolution and Bioagents, Institute of Biology, Unicamp, Brazil; 4 Laboratory of Animal Viruses–Department of Genetics, Evolution and Bioagents, Institute of Biology, Unicamp, Brazil; 5 Clinical Pathology Department, School of Medical Sciences, Unicamp, Brazil; 6 Obstetrics and Gynecology Department, School of Medical Sciences, Unicamp, Brazil; 7 Campinas Department of Public Health Surveillance, Campinas, Brazil; 8 Institute of Infection and Global Health, University of Liverpool, Liverpool, United Kingdom; 9 Faculty of Food Engineering, Unicamp, Brazil; 10 Pediatric Immunology, Center for Investigation in Pediatrics, Faculty of Medical Sciences, Unicamp, Brazil; 11 Neurology Department, Faculty of Medical Sciences, Unicamp, Brazil; 12 Department of Human Development and Rehabilitation, Faculty of Medical Sciences, Unicamp, Brazil

## Abstract

Infection with Zika virus (ZIKV), a mosquito-borne *flavivirus* has been casually linked with increased congenital microcephaly in Brazil from 2015 through 2016. Sensitive and specific diagnosis of patients with Zika fever (ZIKF) remains critical for patient management. We developed a ZIKV NS5 qRT-PCR assay by combining primers described by Balm *et al*. and a new Taqman probe. The assay was evaluated and compared with another assay described by Lanciotti *et al*. (ZIKV 1107) using 51 blood and 42 urine samples from 54 suspected ZIKV patients. ZIKV NS5 performed better in terms of sensitivity with more samples detected as ZIKV-positive (n = 37) than ZIKV 1107 (n = 34) for urine, and ZIKV-positive (n = 29) than ZIKV 1107 (n = 26) for blood. Both assays displayed good overall agreement for urine (κappa = 0.770) and blood (κappa = 0.825) samples. Improved availability of validated diagnostic tests, such ZIKV NS5 qRT-PCR, will be critical to ensure adequate and accurate ZIKV diagnosis.

## Introduction

Zika virus (ZIKV) is an enveloped, positive-sense, single-stranded RNA virus belonging to the *Flaviviridae* family^1^. First isolated in 1947 from a sentinel rhesus macaque in Uganda^2^, ZIKV remained an obscure pathogen until the 2007 outbreak on the Yap Islands in the Federated States of Micronesia^3,4^, followed by a larger epidemic in French Polynesia in 2013 and 2014^5,6^. ZIKV outbreaks subsequently occurred throughout 2014 to 2016 on other Pacific islands^6,7^. In early 2015, ZIKV was identified for the first time in Brazil^8^. Within a year, ZIKV had spread throughout continental South America and into Central America, the Caribbean, and Mexico^8,9^. ZIKV has also re-emerged in Asia^10^, with imported or autochthonous ZIKV infections being reported in Asian countries, including Cambodia, Indonesia, Thailand and Singapore^11^.

Zika infection is an undifferentiated systemic febrile illness with a short-lived fever, non-specific rash, conjunctivitis and arthralgia^3,12^. It is rarely life threatening and maybe sometimes completely asymptomatic^3^. With the spread of ZIKV to the Americas and its association with a marked increase in the incidence of Guillain-Barré syndrome (GBS) in French Polynesia^5,13,14^ and congenital Zika syndrome in Brazil^15–17^, sensitive and accurate diagnosis of patients with ZIKV infection is critical to ongoing epidemiologic surveillance, patient management and eventually treatment of the disease.

In endemic countries such as Brazil, where different arboviruses have been circulating for several years^18^, the detection of ZIKV is more complicated and very challenging. Specifically, the similarity and non-specific nature of the clinical symptoms provoked by these viruses impede correct differential diagnosis during the febrile phase. Moreover, there is a high level of cross-reactivity among different *flaviviruses*^19^. A study done in Rio de Janerio estimated that more than 60% of the Brazilian population are seropositive for dengue virus (DENV)^20^. Furthermore, recent outbreaks of Yellow Fever Virus (YFV)^21^ and the YFV vaccination program in Brazil^22^ have made ZIKV detection by serological tests extremely difficult.

Therefore, the recommended gold standard for ZIKV diagnosis in *flaviruses* endemic areas is molecular-based detection of ZIKV RNA in patients’ specimens during the acute phase of virus infection^23,24^. Several ZIKV reverse transcription PCR (RT-PCR) assays have been reported^23^, and the most well-reported assay is that from the 2007 Yap Islands epidemic^4^. It comprises of 2 one-step real-time RT-PCR (qRT-PCR) reactions, targeting the ZIKV pre-membrane (prM) and envelope (E) genes (referred to as the ZIKV 860 and ZIKV 1107 respectively). In this study, we compared a laboratory-developed qRT-PCR (ZIKV NS5) with ZIKV 1107 using clinical samples collected from 54 suspected ZIKV cases between day 1 and 6 of post illness onset. We analyzed 51 blood samples and 42 urine samples from these suspected ZIKV patients. All of them had symptoms compatible with acute ZIKV infection, including fever, myalgia, rash and conjuctivitis. In addition, urine and blood samples from 20 heathy individuals negative for ZIKV were also include as controls.

## Methods

### Patients

In this study, samples from patients presenting acute febrile disease for less than 7 days from February to June of 2016 were assessed. All patients were admitted to hospitals through their respective emergency departments (ED) at Campinas city, Southeast of Brazil. We recruited a total of 54 ZIKV-suspected patients (42 females, 12 males, mean age 37 years ± 16 years) based on clinical signs, such as the presence of low fever, rash, myalgia and conjunctivitis.

Both whole blood and urine samples were obtained from 39 patients, while only urine was collected from 3 other patients, and only blood from another 12 patients. The negative control group comprised of 20 age-matched healthy individuals without signs of infection within 30 days prior to sample collection. Whole blood and urine samples were obtained from these healthy individuals. Hence in total, 93 clinical specimens from ZIKV-suspected patients together with 40 specimens from negative control individuals were assessed. All samples were tested negative by RT-PCR for DENV.

### Ethics statements

This study was approved by the Research Ethics Committee of the University of Campinas (CEP 053407/2016; CEEA: 56793516.0.0000.54), in accordance with the tenets of the Declaration of Helsinki for human research. Written informed consent was obtained from all patients.

### RNA extraction

Viral RNA from whole blood and urine samples was extracted using the easyMAG® automated extractor (BioMerieux, Quebec, Canada), according to manufacturer’s instructions.

### ZIKV real-time RT-PCR assays

ZIKV 1107 was the reference assay used^25–27^, performed with the following modifications: Cycling conditions were the following: 45 °C for 1 min (RT step); 95 °C for 5 min; 45 cycles of 95 °C for 15 s, and 60 °C for 1 min. ZIKV primers and probe primers were used at final concentrations of 400 nM and 200 nM. In parallel, NS5-targeted qRT-PCR was performed as previously described^28^ with an additional novel lab-designed FAM-labeled molecular beacon probe (TACCAGGAGGAAGGATGTATG) (ZIKV NS5). Cycling conditions and primer/probe concentrations used were similar to that of ZIKV 1107 with the exception of the annealing and extension temperature at the 56 °C for 1 min. Assay exclusivity of ZIKV NS5 was confirmed by testing viral RNA extracted from the following viruses (Supplementary Table 1), and no cross-reactions were identified: Dengue viruses (DENV serotype 1–4), West Nile virus (WNV), yellow fever virus (YFV), chikungunya virus (CHIKV) and o’nyong-nyong virus (ONNV). Analytical sensitivity was also determined using quantitated ZIKV RNA transcripts and the lower limit of detection was estimated as 10 copies for the NS5 gene target. Copy numbers of ZIKV RNA were determined by using the Ribogreen RNA specific Quantitiation Kit (Invitrogen, Carlsbad, USA). RNA transcripts ranging from 2 × 10^6^ to 0.2 copies were performed in pentaplicates to construct standard curves for both qRT-PCR assays to estimate the copy number of ZIKV in patient samples. All qRT-PCR assays were performed on the CFX96 Touch™ Real-Time PCR Detection System (BioRad, Hercules, USA) using QuantiNova Probe RT-PCR Kit (Qiagen, Hilden, Germany) in 25 µL reactions with 3 µL RNA template. qRT-PCRs with cycle threshold (C_t_) values higher than 40 cycles were considered negative.

### Statistical analysis

For statistical analysis, two-tailed Fisher’s exact tests, unpaired t tests, and kappa statistics were performed using Prism 7 software (GraphPad Software Inc, San Diego, USA).

## Results

In the present study, we validated primers described by Balm *et al*. with the addition of a novel probe specific for ZIKV detection using a one-step Taqman qRT-PCR assay (ZIKV NS5). Clinical samples were collated from 54 patients admitted to hospitals facilities in Campinas, São Paulo state, Brazil. These patients had symptoms frequently associated with ZIKV infection, including fever, myalgia, rash and conjunctivitis. The average age of the patients was 37 years ± 16 years (6–65 years) and 77.8% (n = 42) of them were females. The mean period between the onset of symptoms and sampling was 2.92 days (±1.31, ranging from 1 to 6 days). All clinical samples were collected in the first half of 2016, during the rainy season, probably during the first ZIKV outbreak in state of São Paulo, Brazil.

**Figure 1:**
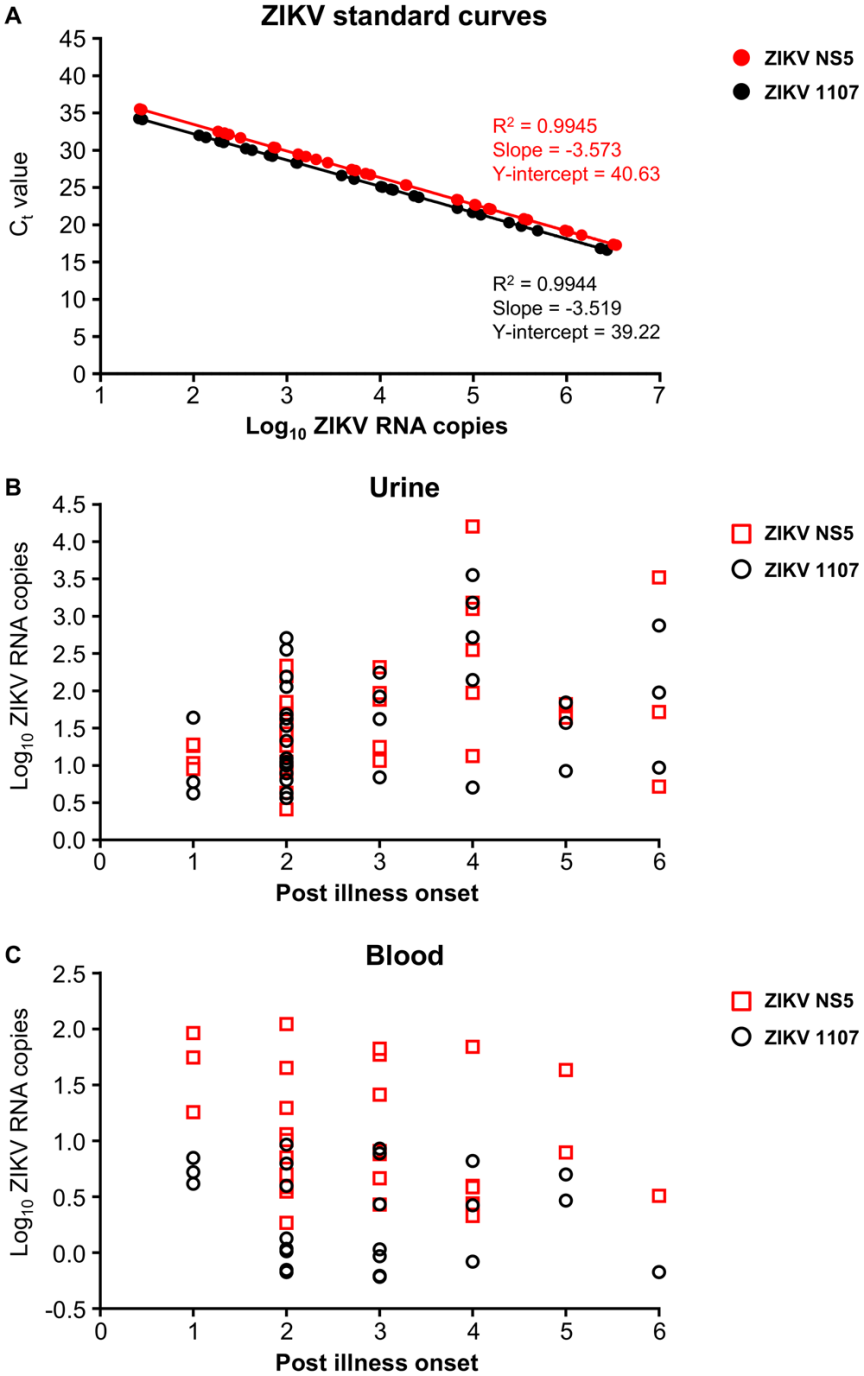
Results of qRT-PCR from patients, Campinas, Brazil, 2016. (**A**) Standard curves of ZIKV NS5 and ZIKV 1107 generated from ZIKV RNA transcripts and extracted ZIKV viral RNA. Comparison between extrapolated ZIKV RNA copies from qRT-PCR of (**B**) urine and (**C**) blood samples with post illness onset.

The sensitivities of ZIKV 1107 and ZIKV NS5 were evaluated and compared using respective standard curves (Fig. 1A) for estimating ZIKV RNA copies in patient samples (Supplementary Tables 2 and 3). ZIKV RNA transcripts with known concentrations were analyzed by both qRT-PCR assays in replicas of 5. Results indicated that these 2 qRT-PCR assays did not differ in their sensitivities (P > 0.05, t tests) (Fig. 1A, Supplementary Figure 1). Both PCR efficiencies, as reflected by the R^2^ values of the respective standard curves (0.9945 for ZIKV NS5 and 0.9944 for ZIKV 1107), were high (R^2^ > 0.90). Of the 54 patients suspected for ZIKV infection, 30 had ZIKV RNA detected in blood and 39 had ZIKV RNA detected in urine using one of the qRT-PCR assays evaluated in this study.

Results for 69 positive samples from ZIKV-suspected patients were stratified by day of post illness onset to examine the pattern of viral load in both urine (Fig. 1B) and blood (Fig. 1C) samples. A relatively high frequency of positive ZIKV detection across one to six days post illness onset was observed for urine (Fig. 1B) when compared to blood (Fig. 1C) samples. ZIKV RNA copies were observed to be highest in urine samples at four days post illness onset. Interestingly, a decreasing trend was seen in blood samples with only one sample showing positive detection at 6 days post illness onset (Fig. 1C). ZIKV RNA copies extrapolated by the respective standard curves (ZIKV 1107 and ZIKV NS5) showed an overlapping range of between 10 to 10^4^ copies for urine samples (Supplementary Table 2, Fig. 1B). However, in blood samples, ZIKV copies extrapolated using the ZIKV NS5 standard curve reflected higher viral copy numbers compared than that from using the ZIKV 1107 curve, with a difference of almost 10 copies (Supplementary Table 3, Fig. 1C). Interestingly, we observed that results from ZIKV NS5 were able to present the trend of viral kinetics in blood samples where the mean of viral load at 1 to 6 days post illness onset were estimated at 33.12, 11.12, 15.86, 10.24, 12.72 and 1.07 copies as compared to 3.28, 1.22, 2.01, 1.36, 3.23 and 0.22 when using ZIKV 1107.

**Figure 2:**
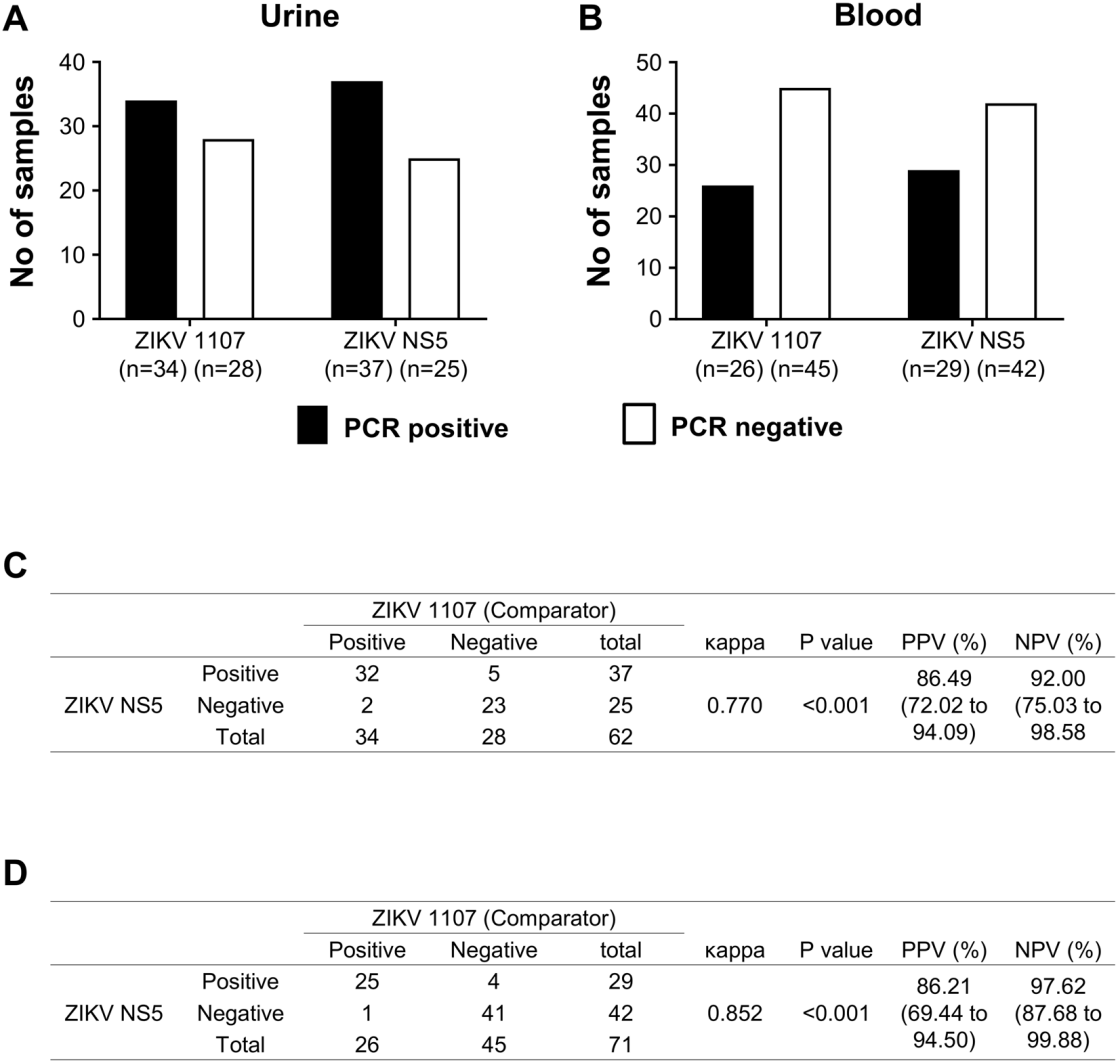
Comparison between ZIKV 1107 and ZIKV NS5. (**A**) Urine and (**B**) blood samples were subjected to ZIKV qRT-PCR detection. Bar-charts show the number of samples which are PCR positive or negative. Using mean C_t_ value = 40 as cut-off, extrapolated ZIKV RNA copies are: ZIKV 1107–0.601, ZIKV NS5–1.497. (**C**) Overall agreement between ZIKV 1107 and ZIKV NS5 was assessed by κappa test for (**C**) urine and (**D**) blood samples. Statistical significance was measured using 2-sided Fisher exact test between the number of samples tested ZIKV positive by ZIKV 1107 or ZIKV NS5. PPV, positive predictive value; NPV, negative predictive value shown with 95% confidence interval.

Samples that were tested ZIKV negative were indicated as ZIKV-PCR negative samples as reflected in Fig. 2C and D. ZIKV detection rates between the 2 qRT-PCR assays were comparable (P > 0.05, Fisher’s test). 34 urine samples were detected as positive with ZIKV 1107, and 37 positive urine samples using ZIKV NS5 (Fig. 2A). Frequency of positive detection by ZIKV NS5 was also higher for blood samples with 3 more samples detected as ZIKV positive (n = 29), compared to ZIKV 1107 (n = 26) (Fig. 2B). The 2 qRT-PCR assays displayed good overall agreement for both urine (κappa = 0.770) (Fig. 2C) and blood (κappa = 0.825) samples (Fig. 2D). The sensitivity and specificity of ZIKV NS5 in comparison to ZIKV 1107 were 94.11% and 82.14% for urine, 96.15% and 91.11% for blood respectively. The positive and negative predictive values were 86.49% and 92.00% for urine samples (Fig. 2C), while 86.21% and 97.62% for blood samples (Fig. 2D).

## Discussion

Low levels of viremia (≤9.26 copies) among ZIKV-infected patients were observed in this study. These estimates were also consistent with that observed in patients from the Yap outbreak (mean quantifiable viremia was 4.4 copies/mL of serum, standard deviation (SD) 0.94)^4^, and patients from the Nicaraguan outbreak (mean quantifiable viremia was 4.7 copies/mL of serum, SD 0.97)^29^. Such low viremia at disease presentation provides a likely explanation for the short window period of less than 5 days for ZIKV detection in blood^4,25,26,30,31^. Future studies with larger numbers of positive specimens will provide further characteristics to the dynamics of ZIKV levels in blood so as to determine if there are correlations of virus load with disease severity and immune responses.

Even though only acute samples of up to 6 days post illness onset were included in the study, ZIKV was still detected at higher titres in urine samples than blood samples of these patients (ZIKV 1107 range was 3.66–3568.61 copies). Moreover, the diagnostic utility of using urine samples was recently confirmed whereby ZIKV RNA was not only detectable in urine at a higher load but with a longer duration than in serum^26,32^. The clinical relevance of testing for ZIKV RNA in urine would allow diagnosis of acute infection after viremia has resolved, extending the window for ZIKV detection^24^. Hence, optimal diagnosis of acute ZIKV infection may require testing of multiple specimen types.

It was observed that quantification by the ZIKV NS5 qRT-PCR reflected lower C_t_ values and therefore higher copy numbers than that from ZIKV 1107 qRT-PCR. It has been reported that in *flavivirus* infection, NS5 expression is higher than that of the E protein^33^. Results also showed that 34 urine samples were detected positive with ZIKV 1107, and 37 positive samples using ZIKV NS5. On the other hand, 29 whole blood samples were detected positive with ZIKV NS5, compared to 26 positive samples by ZIKV 1107. This indicates that the ZIKV NS5 is more sensitive. Therefore, *flavivirus* NS5 could act as a good target for molecular diagnostics for improved sensitivity.

ZIKV diagnostic testing relies on serology and RT-PCR. Immunoglobin (Ig) M antibodies usually appear during the first week after symptom onset, and their appearance is rapidly followed by the appearance of IgG antibodies^4,25^. Although IgM testing may be able to identify recent ZIKV infections, a positive IgM serology still requires confirmation with a plaque reduction neutralization test (PRNT) due to cross-reactivity with other *flaviviruses*, such as DENV^34^. Particularly, serological testing in populations with high DENV exposure and YFV vaccination could be extremely difficult due to cross-reactivity issues. Hence, RT-PCR has an advantage over serology due to its specificity. Additionally, ZIKV RNA detection by RT-PCR is an indication of acute ZIKV infection, although persistence has been observed in urine, saliva, tears and semen^25,31,35–37^.

The worldwide spread of ZIKV and its association with fetal neurologic abnormalities has led to unprecedented interest in the virus^23^. In Brazil, a high percentage of people get concurrent infection with more than one DENV serotype periodically. Thus, people typically assume episodes of rash-febrile illness to be DENV, and only seek medical assistance when critical clinical manifestations appear^38,39^. Since complications generally manifest only after 5 days post illness onset, urine samples are rarely collected. Therefore, the use of this improved qRT-PCR assay will be useful for molecular diagnosis of ZIKV in blood samples. Sensitive and accurate diagnosis of patients with ZIKV infection is critical to ongoing epidemiologic surveillance, management of patients with an undifferentiated febrile illness and assessment of therapies. Importantly, improved availability of validated diagnostic tests, such as the ZIKV NS5 qRT-PCR, will be critical to understand and ensure an adequate, timely and accurate laboratory response.

## Electronic supplementary material

Supplementary Information
