# Supplementary material for: Efficient detection of Zika virus RNA in patients’ blood from the 2016 outbreak in Campinas, Brazil
Source: Sci Rep. 2018 Mar 5;8:4012. doi: 10.1038/s41598-018-22159-2 (PMC5838246; doi:10.1038/s41598-018-22159-2)
Supplement: Supplementary file 1 — Supplementary Information [file 41598_2018_22159_MOESM1_ESM.pdf]

Article submitted to *Scientific Reports* 2018

Efficient detection of Zika virus RNA in patients' blood from the 2016 outbreak in Campinas, Brazil

Improved detection of Zika virus RNA in blood

Carla Cristina Judice[1†], Jeslin J.L. Tan[2†], Pierina Lorencini Parise[3], Yiu-Wing Kam[2], Guilherme Paier Milanez[3], Juliana Almeida Leite[1], Leonardo Cardia Caserta[4], Clarice Weis Arns[4], Mariangela Ribeiro Resende[5], Rodrigo Angerami[5], Eliana Amaral[6], Renato Passini Junior[6]; André Ricardo Ribas Freitas[7], Fabio Trindade Maranhão Costa[1], Jose Luiz Proenca-Modena[3,1*], Lisa F.P Ng[2,8*] and Zika-Unicamp Network[#]

[1]Laboratory of Tropical Diseases – Department of Genetics, Evolution and Bioagents, Institute of Biology, University of Campinas (Unicamp), Campinas, São Paulo, Brazil;

[2]Singapore Immunology Network, Agency for Science, Technology and Research (A*STAR), Singapore;

[3]Laboratory of Emerging Viruses - Department of Genetics, Evolution and Bioagents, Institute of Biology, Unicamp, Brazil;

[4]Laboratory of Animal Viruses - Department of Genetics, Evolution and Bioagents, Institute of Biology, Unicamp, Brazil;

[5]Clinical Pathology Department, School of Medical Sciences, Unicamp, Brazil;

[6]Obstetrics and Gynecology Department, School of Medical Sciences, Unicamp, Brazil;

[7]Campinas Department of Public Health Surveillance, Campinas, Brazil;

[8]Institute of Infection and Global Health, University of Liverpool, United Kingdom

*Correspondence and Address for reprints

Prof. Lisa F.P. Ng, Laboratory of Microbial Immunity, Singapore Immunology Network, Agency for Science, Technology and Research (A*STAR), 8A Biomedical Grove, #04-06, Immunos Building, Biopolis, Singapore, 138648. Tel: +65-64070028, Fax: +65-64642057; E-mail: lisa_ng@immunol.a-star.edu.sg;

Prof Jose Luiz Proenca Modena, Laboratório de Estudos de Vírus Emergentes (LEVE), Departamento de Genética, Evolução e Bioagentes, Instituto de Biologia, Unicamp. Rua Monteiro Lobato, 255 - Campinas - SP - Brasil - CEP 13083-862. Tel: +551935216259; E-mail: jlmodena@unicamp.br

†These authors contributed equally to this work

Supplementary Table 1. List of virus strains used for assay exclusivity

Virus	Strain	Genebank
DENV1	Hawaii	KM204119
	BR/97-409	AF311957
	D1/SG/06K2290DK1/2006	EU081281
	SG(EHI)D1/07771Y14	KJ806959
DENV2	New Guinea C	KM204118
	BR64022	AF489932
	D2/SG/05K4155DK1/2005	EU081180
	NUH_MDC	KU948303
	SG(EHI)D2/09087Y15	KY921905
DENV3	Philippines H87	KU050695
	DENV3-1631	KU509280
	D3/SG/05K4648DK1/2005	EU081225
	D3/H/IMTSSA-SRI/2000/1266	AY099336
DENV4	Philippines H241	KR011349
	8976/95	AY762085
YFV	17D	JX949181
	17D-Tiantan	FJ654700
CHIKV	LR2006_OPY1	DQ443544
	SXM/H-20235-STMARTIN-2013/2003	KX262991
ONNV	IMTSSA/2004/5163	DQ399055

Supplementary Table 2. Results of qRT-PCR of urine samples from patients, Campinas, Brazil, 2016

		ZIKV 1107		ZIKV NS5	
Sample	PIO	mean C_t*	Copies	mean C_t*	Copies
U1	1	37.03	4.20	36.12	18.24
U2	4	26.72	3568.61	29.26	1516.86
U3	4	28.03	1514.47	29.56	1250.22
U4	3	32.45	84.01	33.90	76.28
U5	2	36.40	6.34	36.12	18.24
U6	2	35.71	9.95	36.87	11.25
U7	4	36.74	5.07	36.60	13.39
U8	6	32.26	95.13	34.49	52.15
U9	5	ND	0.00	ND	0.00
U10	6	35.81	9.32	38.07	5.19
U11	2	34.54	21.40	34.96	38.52
U12	2	37.01	4.25	32.78	156.98
U13	2	37.24	3.66	37.36	8.20
U14	3	ND	0.00	ND	0.00
U15	1	33.45	43.67	36.06	18.96
U16	3	31.33	174.81	33.58	93.75
U17	3	ND	0.00	36.83	11.55
U18	3	ND	0.00	ND	0.00
U19	2	ND	0.00	38.36	4.31
U20	3	33.52	41.71	36.88	11.18
U21	2	31.52	154.38	35.60	25.51
U22	2	33.84	33.83	37.18	9.21
U23	2	29.69	511.18	34.75	44.11
U24	2	35.37	12.43	ND	0.00
U25	2	35.62	10.56	36.11	18.36
U26	4	ND	0.00	25.61	15938.01
U27	5	35.96	8.45	ND	ND
U28	1	ND	0.00	36.95	10.69
U29	2	36.07	7.86	35.10	35.20
U30	5	32.74	69.49	34.77	43.54
U31	2	32.00	112.77	34.36	56.71
U32	4	29.66	521.31	33.57	94.35
U33	2	33.30	48.17	34.02	70.60
U34	2	30.24	356.69	32.28	216.65
U35	5	33.69	37.32	34.12	66.19
U36	3	36.27	6.90	36.17	17.66
U37	4	31.67	139.94	31.52	353.56
U38	3	ND	0.00	32.35	207.10
U39	1	36.49	5.98	37.23	8.92
U40	2	35.54	11.12	39.16	2.57
U41	2	33.49	42.54	35.53	26.68

U42	6	29.10	752.01	28.05	3308.07
U43	-	ND	0.00	ND	0.00
U44	-	ND	0.00	ND	0.00
U45	-	ND	0.00	ND	0.00
U46	-	ND	0.00	ND	0.00
U47	-	ND	0.00	ND	0.00
U48	-	ND	0.00	ND	0.00
U49	-	ND	0.00	ND	0.00
U50	-	ND	0.00	ND	0.00
U51	-	ND	0.00	ND	0.00
U52	-	ND	0.00	ND	0.00
U53	-	ND	0.00	ND	0.00
U54	-	ND	0.00	ND	0.00
U55	-	ND	0.00	ND	0.00
U56	-	ND	0.00	ND	0.00
U57	-	ND	0.00	ND	0.00
U58	-	ND	0.00	ND	0.00
U59	-	ND	0.00	ND	0.00
U60	-	ND	0.00	ND	0.00
U61	-	ND	0.00	ND	0.00
U62	-	ND	0.00	ND	0.00

ND, not detectable; C_t, threshold cycle; PIO: Post Illness Onset
* C_t values ≤ 40.0 are positive

Supplementary Table 3. Results of qRT-PCR of blood samples from patients, Campinas, Brazil, 2016

		ZIKV 1107		ZIKV NS5	
Sample	PIO	mean C_t*	Copies	mean C_t*	Copies
B1	3	36.10	7.71	34.30	58.95
B2	2	39.84	0.67	39.67	1.85
B3	2	ND	0.00	ND	0.00
B4	3	ND	0.00	ND	0.00
B5	4	ND	0.00	ND	0.00
B6	1	37.05	4.14	36.14	18.01
B7	1	36.24	7.04	33.61	91.95
B8	4	37.73	2.65	39.45	2.13
B9	4	36.34	6.59	34.05	69.25
B10	3	39.96	0.62	38.25	4.62
B11	2	35.82	9.26	33.32	110.84
B12	2	ND	0.00	ND	0.00
B13	4	39.50	0.83	38.49	3.96
B14	6	ND	0.00	ND	0.00
B15	5	ND	0.00	ND	0.00
B16	6	39.84	0.67	38.81	3.22
B17	3	39.34	0.93	37.48	7.59
B18	2	ND	0.00	38.67	3.53
B19	2	ND	0.00	ND	0.00
B20	3	39.12	1.07	39.09	2.69
B21	2	ND	0.00	ND	0.00
B22	1	ND	0.00	ND	0.00
B23	3	ND	0.00	ND	0.00
B24	2	ND	0.00	ND	0.00
B25	4	39.49	0.84	38.54	3.84
B26	3	ND	0.00	ND	0.00
B27	3	ND	0.00	ND	0.00
B28	2	39.18	1.03	37.58	7.12
B29	2	38.78	1.34	36.84	11.47
B30	2	36.42	6.26	34.72	44.97
B31	2	ND	0.00	38.13	5.00
B32	2	39.11	1.08	37.03	10.15
B33	2	ND	0.00	ND	0.00
B34	4	ND	0.00	ND	0.00
B35	5	37.58	2.93	37.42	7.89
B36	1	36.69	5.24	34.39	55.62
B37	2	39.77	0.70	38.53	3.86
B38	4	ND	0.00	ND	0.00
B39	5	36.76	5.01	ND	0.00
B40	2	37.12	3.96	36.00	19.71
B41	4	ND	0.00	39.05	2.76
B42	2	ND	0.00	38.55	3.81
B43	2	ND	0.00	ND	0.00
B44	3	35.95	8.51	34.11	66.62
B45	5	36.77	4.97	34.79	42.99

B46	3	39.97	0.61	37.38	8.10
B47	3	37.70	2.71	35.58	25.84
B48	1	ND	0.00	ND	0.00
B49	2	ND	0.00	ND	0.00
B50	2	ND	0.00	ND	0.00
B51	6	ND	0.00	ND	0.00
B52	-	ND	0.00	ND	0.00
B53	-	ND	0.00	ND	0.00
B54	-	ND	0.00	ND	0.00
B55	-	ND	0.00	ND	0.00
B56	-	ND	0.00	ND	0.00
B57	-	ND	0.00	ND	0.00
B58	-	ND	0.00	ND	0.00
B59	-	ND	0.00	ND	0.00
B60	-	ND	0.00	ND	0.00
B61	-	ND	0.00	ND	0.00
B62	-	ND	0.00	ND	0.00
B63	-	ND	0.00	ND	0.00
B64	-	ND	0.00	ND	0.00
B65	-	ND	0.00	ND	0.00
B66	-	ND	0.00	ND	0.00
B67	-	ND	0.00	ND	0.00
B68	-	ND	0.00	ND	0.00
B69	-	ND	0.00	ND	0.00
B70	-	ND	0.00	ND	0.00
B71	-	ND	0.00	ND	0.00

ND, not detectable; C_t, threshold cycle; PIO: Post Illness Onset
* C_t values ≤ 40.0 are positive

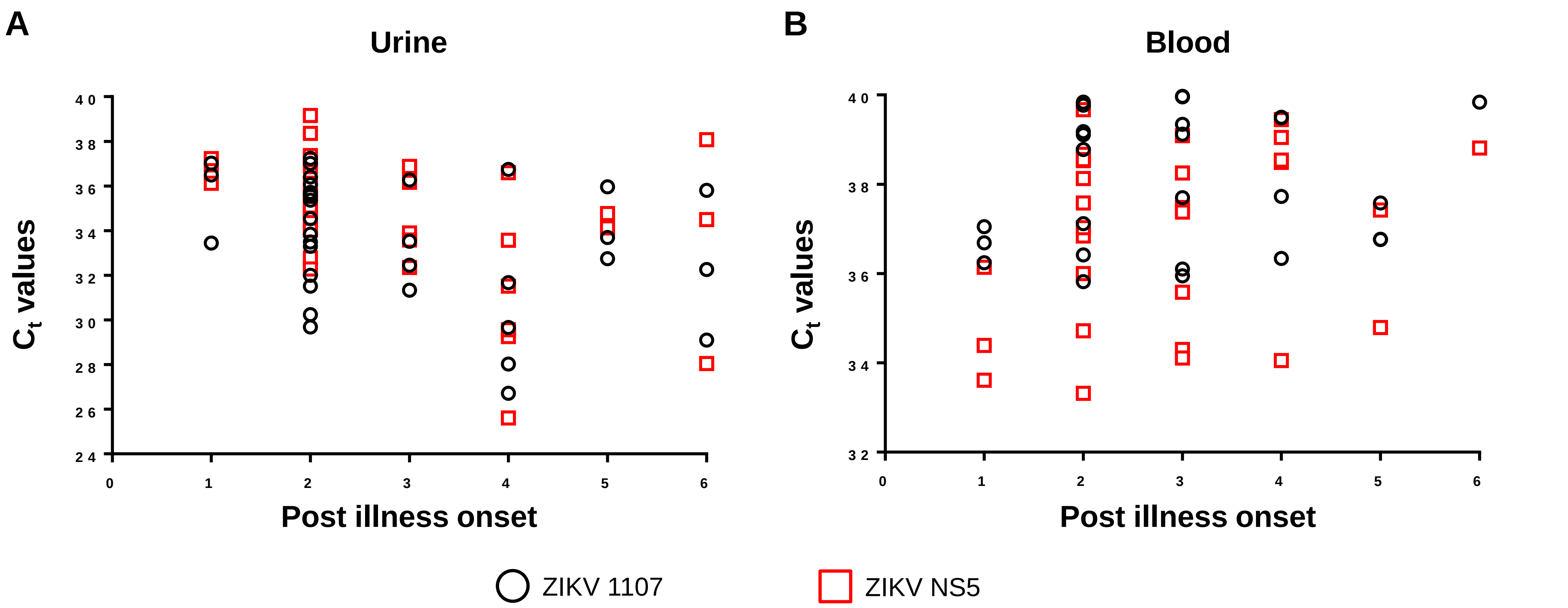

Supplementary Figure 1. Urine (n=62) and blood (n=71) samples were subjected to ZIKV qRT-PCR detection using ZIKV 1107 and ZIKV NS5. Comparison between C_t values from qRT-PCR of (A) urine and (B) blood samples with post illness onset.

Supplementary Figure 1 Judice & Tan et al., 2018
